# London as a Surgical Teaching Centre

**Published:** 1906-09-01

**Authors:** 


### LONDON AS A SURGICAL TEACHING CENTRE.

Early London.

It is not easy at first sight to discover the causes which have led to the pre-eminence of one city over another to such an extent as to make it the capital of a country. The capital is synonymous with the seat of government in most instances, but not always, as in the case of Washington and New York. Sometimes the will of a despot has made the capital what it is and has placed it on a given site as in the towns of St. Petersburg and Madrid. In other cases the accident of situation on a navigable river and at a secure distance from its mouth so that it has not been subject to raids, this made Paris and London. But Paris did not always represent the capital of France, nor London of England. Winchester was the capital of England at least as late as 1187. The King kept his exchequer there so literally that he provided a watchman to look after his treasure and made him an allowance of candles during the dark days of winter to enable him to see that nothing was stolen.

### Some Causes of its Greatness.

There must be other causes therefore than the Royal will or the mere accident of place to enable some towns to become capitals to the exclusion, or even the supersession, of others apparently as well placed. The true reason appears to be the character of the inhabitants. It may well be said that the same character cannot persist for ages through the shifting multitudes which compose such a population as that of London. But a little thought shows that the corporate type does not change much though the individuals composing it may vary greatly. No one can have taken part in a large representative meeting without marvelling how important results are obtained by seemingly trivial changes and the mere verbal alterations of different persons until at last broad conclusions of substantial accuracy and meeting the wishes of the majority are eventually obtained. But the conclusions would differ if the meetings were held in different towns, though they might be substantially in accord, for the Birmingham man does not think quite on the same lines as the inhabitant of Liverpool, nor he of Bristol like the dweller in Newcastle; whilst the Scotch, Irish, and Welsh are in many respects wholly distinct. London has always been sturdy, so long ago as the Conquest, its citizens succeeded in wringing from William the King the short and comprehensive charter which secured its liberties against all his successors. " I will," it runs, " that you be lawworthy as you were in King Edward's day. And I will that every child be his father's heir after him, and I will not endure that any man offer any wrong to you." The charter gave all that could be wanted, but it required a strong body of men to wrest it from a Conqueror at a time when public opinion went for nothing. Thus the London citizens were strong in the beginning; strong and clear-sighted they still remain, and they have behind them the venerable traditions of many centuries in surgery as in law and in politics.

### Barber-Surgeons.

Medical teaching was in the hands of City companies for many years. The teaching of surgery in particular was the business of the Barber-Surgeon's Company. Similar bodies existed in Exeter, Norwich, Oxford, Newcastle, and perhaps in many other towns. These provincial bodies modelled themselves upon the London Corporation, copied their by-laws, and essayed to do a similar work in a smaller way. Occasionally they succeeded for a time, but sooner or later in every case they were destroyed by trade rivalry and internecine feuds. In London itself the separation of the barbers and the surgeons took place in 1745, but fortunately the disruption occurred at the very moment when a small body of energetic surgical teachers was beginning to show how much students had to learn. These teachers soon attracted to themselves students from all parts of England, brought them in large numbers " to walk the hospitals " in London, and thus gave the metropolis a monopoly of surgical teaching. At first and for many years anatomy was the sole means of instruction and the great surgeons of the pre-anæsthetic days were all first-rate practical anatomists. By no other means could they perform operations when it was of the first importance to cut as quickly as possible and yet to avoid all important structures. Surgery was taught by lectures, and clinical teaching was still in its infancy. With the advent of John Hunter came morbid anatomy, and his genius invented the principles of surgery and thus made a science of what had hitherto been a mere handicraft. Hunter's value was soon recognised. He had a following of students who became the most distinguished teachers of the next generation, not in London only, but throughout England and in the United States of America.

### Early Examination.

At the end of the eighteenth century it was necessary for any surgeon to be examined before he began to practise unless he were going to live in a University town or in London and its immediate neighbourhood. It is very creditable, therefore, that so many students came to London to study and afterwards presented themselves for examination at the Surgeons' Company or at the Society of Apothecaries. The requirements of the Apothecaries Act passed in 1815 compelled every student to submit himself to a qualifying examination, and as English students were compelled to do this in London, medical teaching became almost a monopoly of the metropolis though there were good teaching centres in Manchester, Newcastle, Norwich, and other towns. At this time students depended largely upon private teaching, conducted by a class of men known as " grinders," for the era of clinical work was not yet, nor was any practical examination required by the examiners. The difficulties of travelling and the general inertia of the middle of the last century prevented any continuous acquisition of knowledge.

### The First Post-Graduate Course.

When a student had qualified he settled for the most part where he intended to spend the rest of his life and soon became part and parcel of the neighbourhood, taking few holidays and spending even those in hunting, shooting, or fishing. He was content, therefore, to learn by his own experience, and had no desire to make himself more widely acquainted with the advance of professional work than could be obtained by reading the *Lancet*. Local medical societies hardly existed except in the larger towns, and the only post-graduate course attempted was in the Edgware Road, where it soon proved abortive. But it should not be forgotten that the whole of modern medical teaching sprang out of a post-graduate class, for Dr. William Hunter took over from William Sharp, of Guy's, a class of Navy surgeons which used to meet in Covent Garden during the winter afternoons, and from this class rose the Hunterian or Windmill Street school of medicine.

### Development of Clinical Teaching.

Clinical teaching was developed in the wards of the great London hospitals by such men as Sir

Astley Cooper and Sir Benjamin Brodie; in the out-patient rooms by Abernethy and Sir James Paget. Their methods, chiefly Socratic, are still employed with the greatest advantage, and their tradition yet lingers. The wealth of clinical material is indeed the great glory of London; it appears inexhaustible and is ever varied. The number of cases which he sees makes it well worth the while of every medical man to spend some part of his time in the metropolis either as an advanced student or as a post-graduate if he is unable to take out the whole course. He will not only have an unrivalled opportunity of seeing cases from all parts of the world, but he will be able to work amongst some of the finest pathological collections of Europe. The Hunterian Museum in Lincoln's Fields, the museums of St. Bartholomew's, St. Thomas's, Guy's, the London and University College, are magnificent and are never final.

### Cosmopolitan Gain.

But if a student wishes to take full advantage of his stay in London he should attach himself to one of the larger hospitals. He will find there an *esprit de corps* and a camaraderie which it is difficult for an outsider to realise. The bias, indeed, is not altogether good, but it is and ought to be corrected afterwards by greater experience and a wider outlook. In the larger schools men soon lose all provincialism and gain the cosmopolitanism, or power to appreciate the value of knowledge from whatever source it may come, which is the true criterion of the highest form of culture. In a large school, too, men are brought into intimate and daily contact with comrades from all parts of the world, they see the idlest and are able to measure their strength with the ablest and the most ambitious. Their teachers are men of world-wide reputation who are seeking to enlarge the bounds of knowledge. They learn from them enthusiasm, truth, and humility, for all the great workers in science are but advanced students conscious of repeated mistakes and yet driven by the love of their work to renewed efforts. The professorial tone has long left the medical schools of London, and no one now speaks *ex cathedrâ*, for everything is submited tó the test of experience and observation.

### Various Sources of Medical Learning.

To an earnest student bent upon original work London presents other attractions besides the medical schools. The wealth of clinical material is so great that much of it runs to waste because it cannot be properly husbanded. The large workhouse infirmaries should form a most valuable store for anyone who is at work on a particular subject. The asylums of the London County Council are not used systematically to the extent of their resources, and there are many special hospitals in London where a student hardly ever enters. On the other hand, as a recent series of articles in our pages shows, there are abundant facilities for graduate teaching in the London hospitals, for in no town are there more hospitals devoted to the eye, throat, diseases of children, or orthopædics, where there is such an abundance of teaching, of teachers, and of students. During the last year, too, the rearrangement of the Dreadnought hospital has placed the teaching of tropical medicine in London upon a secure footing.

### The Medical Societies.

The medical societies of the Metropolis are both good and numerous. Several of them are provided with excellent libraries from which members can borrow books for home use. A committee is working at the present time to ascertain whether it is possible to amalgamate the chief medical societies into an Academy of Medicine such as has long been desired by those who have at heart the best interests of medical science in London. Many difficulties have to be overcome and the progress is necessarily slow, but it is hoped that an academy of medicine will eventually be established.

### Laboratory Work.

The work of the junior student in medicine is as well provided for in London as is the teaching of the advanced student. The ideal method is undoubtedly to allow all the preliminary work to be done at centres where the student can easily obtain fresh air and the exercise which is necessary for him while he is growing. He is then able to assimilate more easily what he is taught, and he can at the same time lay up a stock of health against the time when the greater part of his existence is spent amongst out-patients and in the wards of a hospital. The magnificent laboratories which have been built at Oxford and Cambridge as well as in many of the provincial towns, make provision for these needs, and they far exceed in size and equipment anything which exists at present in the metropolis. The time appears to be at hand, however, when the London schools of medicine will agree to allow of the teaching of anatomy and physiology at a central institute independent of them all; whilst the chemistry, physics, and biology necessary to understand rightly the institutes of medicine will be taught at school to those boys who intend to follow the profession of medicine. Pathology is so intimately blended with the daily requirements of the physician and surgeon that each hospital will be obliged to maintain a separate pathological staff, but in the Lister Institute there is already provided a splendid centre for those who wish to enter upon original research.

### The Best Intellects in Surgery.

It will be seen from this short review that the glamour of the capital has attracted to it the best intellects in medicine, just as it has done in law, in finance, and in business. Many individuals of equal, if not of superior attainments, are to be found in other towns, but the average is undoubtedly higher in London than elsewhere in England. It is good for students to associate with such men, though it may only be for a time. They learn from them lessons of integrity, of zeal, and of perseverance which not only raise their own standard of life, but, by their influence, improve the general tone of the medical profession wherever it is subsequently their lot to settle. For all students the wards, operating theatres, and pathological museums are the proper places in which to learn surgery. The junior student should supplement the knowledge thus gained by a diligent attendance at the operative surgery classes. The senior student will do well to take out a post-graduate course and go to meetings of the societies, especially on the nights when cases are shown. He will thus have an opportunity of seeing some of the rarer forms of disease, and he will have the opportunity of following the discussion which attends their exhibition. In like manner some of the hospitals, notably St. Bartholomew's Hospital, on Thursdays, have instituted a series of "consultations," at which the more difficult surgical cases are shown and discussed, more especially with regard to diagnosis and treatment. The student learns at such gatherings not only the methods of examination, but also the very different manner in which the same facts appeal to different trained intellects, and he is able to observe how the conservatism of one surgeon is tempered by the zeal of another or *vice versa.*

It is advisable, therefore, for a student to have done with his preliminary studies as quickly as the exigencies of examining boards will permit. He should then concentrate his attention on the clinical side of his work attending the wards and out-patient room and taking every opportunity of handling patients with a view to diagnosis, bearing in mind the golden rule of the Cambridge School of Medicine: "Eyes first and much, hands next and least, tongue not at all." Every moment of his spare time should be spent in the pathological museum, for there alone can he find a true explanation of the many problems awaiting solution in the wards and an intimate knowledge of morbid anatomy and of pathology in its broadest sense will enable him to forecast the series of events in the more difficult as well as in the easier cases. When the hospital museum has been learnt thoroughly the student should not neglect to enlarge his knowledge by frequent visits to the Royal College of Surgeons, where the Hunterian Museum contains the most magnificent pathological collection in the world.

### The Question of Expense.

The question of expense hardly enters into consideration. The medical profession is still one of the cheapest into which a son can be put, and it affords a certainty of a livelihood to everyone who is steady and in good health. The total fees for the five years' course amounts to 165 guineas at the larger hospitals, and it is greatly reduced for those who have already taken out a part of their training at one of the Universities; whilst for ten or twenty guineas special courses can be taken out in medical or surgical practice by those who have already qualified elsewhere. Living in London is as cheap or as expensive as one likes to make it, but the large medical schools in the metropolis now have a college under the supervision of a warden charged to look after the interests and the expenses of the students who live in it.

